# Frequency and correlates of poor glycemic control in patients with type 2 diabetes at Jimma Medical Centre, Ethiopia: a cross-sectional study

**DOI:** 10.11604/pamj.2024.47.7.37452

**Published:** 2024-01-08

**Authors:** Misganaw Asmamaw Mengstie, Endeshaw Chekol Abebe, Tadesse Asmamaw Dejenie, Mohammed Abdu Seid, Assefa Agegnehu Teshome

**Affiliations:** 1 Department of Biochemistry, College of Medicine and Health Sciences, Debre Tabor University, Debre Tabor, Ethiopia,; 2 Department of Biochemistry, College of Medicine and Health Sciences, University of Gondar, Gondar, Ethiopia,; 3 Department of Physiology, College of Medicine and Health Sciences, Debre Tabor University, Debre Tabor, Ethiopia,; 4 Department of Anatomy, College of Medicine and Health Sciences, Debre Tabor University, Debre Tabor, Ethiopia,

**Keywords:** Type diabetes mellitus (DM), glycated hemoglobin, glycemic control, metabolic risk factors

## Abstract

**Introduction:**

the majority of studies in Ethiopia determine the prevalence of glycemic control employed by fasting blood sugar (FBS), which is impacted by a variety of factors. Hence, the purpose of this study was to assess the status of glycemic control using HbA1c and its correlates in patients with type 2 diabetes in Southwest Ethiopia.

**Methods:**

a cross-sectional study was employed among 124 T2 diabetes mellitus (DM) patients at Jimma Medical Center (JMC), Southwest Ethiopia. HbA1c and FBS were estimated using the Cobas 6000 analyzer. The body mass index (BMI) and waist-to-hip ratio were calculated as the standard formula. Data were analyzed by SPSS version 25. Logistic regression analysis was employed to identify independent risk factors associated with poor glycemic control of DM patients.

**Results:**

males comprised 63.7% (n=79) of the total respondents. The mean age of aOR: 2.21, 95% CI 1.13, 4.34; p = 0.01f participants was 51.84 ± 11.6 years; 60.5% (n=75) of T2 DM patients were in poor glycemic control (HbA1c ≥ 7%). In multivariate logistic regression analysis, BMI of ≥ 30, (aOR: 2.21, 95% CI 1.13, 4.34) increased waist-to-hip ratio (aOR: 1.63, 95% CI 0.82, 2.18), high systolic blood pressure (aOR: 1.52, 95% CI 1.11, 6.23), high FBS (aOR: 1.61, 95% CI 1.00, 4.12), and longer duration of DM (aOR: 1.23, 95% CI 0.87, 1.88) were associated with poor glycemic control.

**Conclusion:**

the level of poor glycemic control in the study population is high. Obesity and/or overweight, central obesity, systolic hypertension, and fasting blood sugar levels were all associated with poor glycemic control in T2 DM patients.

## Introduction

Diabetes mellitus (DM) is a serious, chronic disease that occurs either when the pancreas does not produce enough insulin or when the body cannot effectively use the insulin it produces [1]. Long-term hyperglycemia in DM may cause damage to various organs and can lead to the development of disabling and life-threatening complications, such as cardiovascular disease, nephropathy, neuropathy, and retinopathy [2]. Diabetes is increasing at an alarming rate throughout the world and about 80% of patients with diabetes live in low and middle-income countries [3].

In Ethiopia, the prevalence of diabetes is estimated to be in the range of 2-3% and the number of people with diabetes mellitus in the year 2000 was approximately 800,000 and the number is expected to escalate to 1.8 million by the year 2030 [4].

Glycemic control is the most important predictor of DM-related complications and deaths. In Ethiopia, only one-third of patients with diabetes were achieving good glycemic control based on fasting plasma glucose measurements [5]. Results from many observational and randomized controlled trials have shown that, strict control of glycemic level helps to prevent long-term complications associated with DM [6-9]. However, achieving good glycemic control remains a challenge in patients with diabetes [10]. Estimating glycated hemoglobin (HbA1c) is the standard method for assessing long-term glycemic control in DM patients. The HbA1c is a hemoglobin variant, formed by condensation of a glucose molecule with N-terminal residue in the β-chain of hemoglobin. The binding is non-enzymatic and irreversible until the end of the red blood cells (RBCs) lifespan [11]. Analysis of HbA1c in the blood provides the average blood glucose levels of DM patients during the past 2-3 months, which is the expected life span of RBCs. Thus, the level of HbA1c depends on the concentration of blood glucose [12]. HbA1c is the most effective method of glycemic monitoring than other glucose-based measurements [13].

The major goal of therapeutic intervention to reduce diabetes-related mortality and morbidity is to maintain a good glucose level for all DM patients. Despite this, a large percentage of people with diabetes around the world have uncontrolled diabetes [8]. Furthermore, obesity or overweight, high blood pressure, high fasting blood sugar (FBS), and high blood triacylglycerol levels all contributed to a substantial proportion of non-communicable disease-related mortality and disability, including diabetes [14]. The majority of studies in Ethiopia to determine the prevalence of glycemic control employed FBS, which is impacted by factors such as food intake, stress, exercise, and acute treatment reactions [15-17]. In addition, previous studies have not looked into the link between poor glycemic control and metabolic risk factors in patients with diabetes. Hence, the purpose of this study was to assess the status of glycemic control using HbA1c and its correlates in patients with type 2 diabetes at Jimma Medical Center (JMC), Southwest Ethiopia.

## Methods

**Study design and setting:** a cross-sectional study design was carried out among 124 T2 DM patients in JMC, southwest Ethiopia, from July 27 to August 30, 2020. JMC is one of the teaching hospitals in the country, located about 353 km from Addis Ababa, the capital city of Ethiopia. The hospital is a specialized and referral hospital that provides services for more than 15 million people in its catchment area. JMC has a chronic illness follow-up unit where chronic diseases such as DM patients are regularly monitored and treated.

### Study population

**Inclusion criteria:** all confirmed patients with T2 DM on follow-up care at JMC were included in the study.

**Excursion criteria:** patients who had anemia, chronic diseases, any type of malignancy, or recurrent malaria infection were excluded from the study after being verified by asking and critically checking their medical records.

**Sample size determination and sampling technique:** the minimum sample size was calculated by using Epi Info software version 7.2 StatCalc. By considering the expected frequency of poor glycemic control in T2 DM patients conducted in west Shewa Zone, Ethiopia using HbA1c which is 63.8% [16], 95% of confidence level, 5% of acceptable margin of error, and population size (number of T2 DM patients on follow up care in JMC) of 2,700. The final sample size becomes 208. However, due to resource limitations, only 124 study participants were enrolled in the study through a consecutive sampling technique. Since this study was part of the previous study conducted by Asmamaw *et al*. [18], the study population share similarity.

**Study variables:** poor glycemic control as measured by HbA1c ≥ 7% was the dependent variable. The independent variables were metabolic risk indices such as Body Mass Index (BMI), Waist to Hip-ratio (WH-R), Systolic Blood Pressure (SBP), Diastolic Blood Pressure (DBP), recent FBS, regular physical exercise, and duration of DM.

**Data collection:** questionnaires, anthropometric measures, record reviews, and blood sample analysis were used to collect data. Two bachelor of science (BSc) nurses working in a chronic illness follow-up clinic collected data from the questionnaire, anthropometric measures, and records, whilst laboratory technologists in collaboration with the principal investigators carried out blood sample collection and test analysis.

**Questionnaire:** an interviewer-administered structured questionnaire adapted from the WHO STEPS instrument [19], was used to collect socio-demographic profiles, behavioral related factors, and clinical data of eligible participants. In order to keep its consistency first, the questionnaire was prepared in English and translated to both Amharic and Afan Oromo local languages then back-translated to English by multilingual experts.

**Anthropometric and blood pressure measurements:** anthropometric measurements for body weight (kg), height (cm), waist (cm), and hip (cm) circumference were made for all patients according to the WHO recommendation. Then the Body Mass Index (BMI) was then calculated using a standard formula as weight (kg)/height (m^2^). Underweight (18.5 kg/m^2^), normal (18.5-24.9 kg/m^2^), overweight (25-29.9 kg/m^2^), and obese (30 kg/m^2^) are the four BMI categories identified by the WHO. The waist-to-hip ratio (WH-R) was calculated by dividing waist circumference by hip circumference. After a 5-minute rest in a sitting position, systolic (SBP) and diastolic (DBP) blood pressure were taken using a conventional adult arm cuff mercury sphygmomanometer. Two readings were taken at five-minute intervals, and the average of the two was recorded as the participant's final blood pressure.

### Laboratory analysis

**Blood sample collection:** immediately after the interview and anthropometric measurements, three milliliters (3 ml) of the venous blood sample was collected from each eligible study participant using 5 cc sterile syringes, through the aseptic technique. The blood samples were collected into a labeled test tube containing ethylene-diamine-tetra acetic acid (EDTA) anticoagulant. All blood tubes were labeled with the participant´s unique number. The specimen then was transported to the JMC laboratory unit for analysis on the same date of specimen collection to prevent whole blood hemolysis.

**HbA1c and FBS measurement:** fully automated Cobas® 6000 series chemistry analyzer (Roche Diagnostic, Germany) was used to determine the blood level of HbA1c and FBS.

**Operational definitions:** 1) Poor glycemic control: was defined as an HbA1c level ≥ 7% according to the American diabetes association (ADA) standards of medical care in diabetes 2021 recommendation [20]; 2) regular physical exercise: participants who purposively perform any kind of exercise for over 30 minutes at least 3 times per week.

**Statistical analysis:** data were entered into statistical software Epi-data window version 3.1 and exported to SPSS software version 25 for analysis. All variables were cleaned through running frequencies to avoid missing values. Descriptive statistics (mean and standard deviation for continuous variables; and frequencies and percentages for categorical variables) were computed. The level of glycemic status was determined by categorizing the study participants into poor and good glycemic control based on their HbA1c values. To determine the crude relationship between the factors that contribute to poor glycemic control, binary logistic regression was used. Then, variables that were significant at P < 0.25 were added to the final multivariate model to identify significant factors that could independently predict poor glycemic control in T2 DM patients. Both crude (COR) and adjusted odds ratios (AOR) together with their corresponding 95% confidence intervals were computed to assess the strength of association between the outcome and independent variables. In the multivariate analysis, variables with a p-value of less than 0.05 were considered significant. In the final model, the Hosmer-Lemeshow test was used to determine model fitness, and a value greater than 0.05 was considered a good fit. Finally, the result was presented in texts and tables.

**Data quality management:** accuracy, clarity, and completeness of data were reviewed and checked on a daily basis by the supervisors. Sample collection, handling, processing, and analysis were performed by strictly following Standard Operational Procedures (SOPs). Moreover, all reagents used were checked for their expiry date and the instrument was calibrated every day by running quality control samples before the actual sample test according to the manufacturer´s recommendation.

**Ethical considerations:** the Institutional Review Board (IRB) of Jimma University provided ethical clearance and approval (reference number: IHRPG1/716/20). After a thorough explanation of the study's objective, benefits, and procedures, each study participant signed a written informed consent form. Instead of using personal identification, participants' information was kept confidential by employing unique codes and medical record numbers. The study was carried out per the Helsinki Declaration. Furthermore, each study participant's samples were only used for the reason intended. Abnormal laboratory findings of the study participants were reported to the attending health care experts for further management during their follow-up.

## Results

**Socio-demographic characteristics of study participants:** a total of 124 T2 DM patients participated in this study. Out of the total respondents, 79 (63.7%) of them were males. The mean (± SD) age of study participants was 51.8 (± 11.6) years ranging from 30 to 83 years. The majority (44.4%) of the respondents were found in the age group of 45 to 59 (Table 1).

**Table 1:** sociodemographic characteristics of respondents in Jimma Medical Centre (JMC), Southwest Ethiopia, 2020

Variables	Category	Frequency (n)	Percentage (%)
Sex	Male	79	63.7
	Female	45	36.3
Age in year	30-44	35	28.2
	45-59	55	44.4
	60-74	29	23.4
	≥75	5	4
	Mean (±SD)	51.8 (11.6)	
Marital status	Single	5	4
	Married	106	85.5
	Divorced	6	4.8
	Widowed	7	5.6
Educational status	No formal education	29	23.4
	Primary	45	36.3
	Secondary	24	19.4
	Above secondary	26	21
Monthly income (ETB)	≤ 1,000	28	22.6
	[1,001-3,000)	61	49.2
	≥3,000	35	28.2
Residence	Urban	72	58.1
	Rural	52	41.9
Family history of DM	Yes	29	23.4
	No	95	76.6

ETB: Ethiopian birr; DM: diabetes mellitus; SD: standard deviation

**Anthropometric and clinical characteristics of study participants:** the overall average BMI of the study participants was 24.1 kg/m^2^. About 26.6% and 7.3% of T2 DM patients were overweight and obese respectively. The result also showed that about 12.1% of T2 DM patients had SBP of more than 140 mmHg. The mean duration of diabetes mellitus since diagnosis was found to be 6.3 ± 3.7 years. The overall mean of FBS and HbA1c was 150.26 ± 59.2 mg/dl and 7.99 ± 1.77% respectively (Table 2).

**Table 2:** anthropometric and clinical features of respondents in Jimma Medical Centre (JMC), Southwest Ethiopia, 2020

Variables	Category	Frequency (n)	Percentage (%)
BMI (kg/m^2^)	<18.5	6	4.8
	(18.5-24.9)	76	61.3
	(25-29.9)	33	26.6
	≥ 30	9	7.3
	Mean (±SD)	24.1 (±3.8)	
WH-R	Mean (±SD)	0.94 (±0.09)	
SBP (mmHg)	< 140	109	87.9
	≥ 140	15	12.1
	Mean (±SD)	127.8 (±15.7)	
DBP (mmHg)	< 90	119	96
	≥ 90	5	4
	Mean (±SD)	79.5 (±10.1)	
Duration of DM (year)	≤ 5	59	47.6
	(6-10)	52	41.9
	≥ 10	13	10.5
	Mean (±SD)	6.3 (±3.70)	
Recent FBS (mg/dl)	Mean (±SD)	150.26 (±59.2)	
HbA1c (%)	Mean (±SD)	7.99 (±1.77)	
Regular exercise	Yes	34	27.4
	No	90	72.6

BMI: body mass index; DBP: diastolic blood pressure; FBS: fasting blood sugar; SBP: systolic blood pressure; SD: standard deviation; WH-R: waist to hip ratio

**Glycemic status of study participants:** the glycemic control status of study participants was determined through HbA1c measurement. Based on the level of HbA1c the total number of T2 DM patients was categorized into good glycemic control (HbA1c <7%) and poor glycemic control (HbA1c ≥7%). Accordingly, 75 (60.5%) of T2 DM patients were found in poor glycemic control.

**Factors associated with poor glycemic control of study participants:** to identify factors associated with poor glycemic control among T2 DM patients, bivariate and multivariable logistic regression analyses were performed. On binary logistic regression analysis, age, BMI, WH-R, SBP, DBP, duration of DM, regular physical exercise, and FBS were significantly associated with poor glycemic control at a p-value of < 0.25. However, in the final multivariate logistic regression model, five factors emerged significantly associated with poor glycemic control (Table 3). Obesity (aOR: 2.21, 95% CI 1.13, 4.34; p=0.01), high SBP level (aOR: 1.52, 95% CI 1.11, 6.23; p=0.02), patients with duration of DM ≥10 years (aOR: 1.23, 95% CI 0.87, 1.88; p=0.04), and FBS (aOR: 1.61, 95% CI 1.00, 4.12; p=0.005) were significantly associated with poor glycemic control at a p-value of 0.05.

**Table 3:** poor glycemic control and its association with metabolic risk factors among respondents in Jimma Medical Centre (JMC), Southwest Ethiopia, 2020

Variables	Category	Glycemic status		cOR (95% CI)	P-value	aOR (95% CI)	P-value
**PGC**	**GGC**
Age (in year)	Continues	75	49	0.88 (0.74, 1.05)	0.17	0.90 (0.77, 1.06)	0.21
BMI (kg/m^2^)	<18.5	4	2	0.98 (0.27, 3.32)	0.15	0.42 (0.05, 3.55)	0.32
	(18.5-24.9)	44	32	1		1	
	(25-29.9)	20	13	1.37 (0.99, 7.04)	0.09	1.32 (0.15, 6.82)	0.07
	≥ 30	7	2	1.83 (0.17, 10.23)	0.14	2.21 (1.13,4.34)	0.01^*^
WH-R	Continues	75	49	2.53 (1.05, 8.76)	0.11	1.63 (0.82. 2.18)	0.03^*^
SBP (mmHg)	< 140	66	43	1		1	
	≥ 140	9	6	1.44 (0.39, 5.27)	0.18	1.52 (1.11, 6.23)	0.02^*^
DBP (mmHg)	< 90	73	46	1		1	
	≥ 90	2	3	1.08 (0.31, 8.87)	0.20	1.07 (0.14, 9.13)	0.09
Duration of DM (in year)	≤ 5	34	25	1		1	
	(6-10)	34	18	0.79 (0.48, 1.29)	0.42	0.99 (0.93, 1.06)	0.22
	≥ 10	7	6	1.13 (0.11, 1.32)	0.09	1.23 (0.87, 1.88)	0.04^*^
FBS (mg/dl)	Continues	75	49	1.52 (1.00, 3.03	0.01	1.61 (1.00, 4.12)	0.005^*^
Regular exercise	Yes	20	14	1		1	
	No	55	35	1.36 (0.09, 3.23)	0.18	1.03 (0.99, 1.08)	0.14

* : significant at a p-value of < 0.05 in multivariate logistic regression analysis; GGC: good glycemic control; PGC: poor glycemic control; 1: reference category

## Discussion

The cornerstone of managing T2 DM and preventing its associated complications is maintaining good glycemic control [21]. One of the challenges for the rising burden of T2 DM-related complications is poor glycemic control [22]. The prevalence of poor glycemic control and its contributing factors were examined in the current study.

In this study, the prevalence of poor glycemic control among T2 DM patients is 60.5%. The finding is lower than the studies conducted in different parts of Ethiopia [10,23-25]. The disparity may result from variations in how patients' glycemic status is assessed. All of these investigations employed FBS to assess the patient's glycemic status, which could be impacted by a number of factors. However, in our study, we used the more reliable method called HbA1c to determine the level of glycemic status in DM patients. The finding is also lower than the studies conducted in Saudi Arabia and Morocco reported as 74.9 % [26] and 66.3% [27] respectively. The possible justification for the discrepancy may be due to the differences in the study population, sample size, and the method used to assess the glycemic level. On the other hand, the result of this study is comparable with other studies conducted in Nekemte, Ethiopia, 59.5%, [28], Gondar, Ethiopia, 60.5% [29], and Mekele, Ethiopia, 61.9% [30].

In the current study, logistic regression analysis was used to evaluate the association between glycemic status and metabolic risk indices such as BMI, WH-R, blood pressure, FBS, and exercise. Accordingly, obese and/or overweight T2 DM patients have higher odds of poor glycemic control in comparison to patients with a normal BMI. The likelihood of poor glycemic control in the study participants is also high as the level of WH-R is increased. This is consistent with the majority of earlier studies carried out in Ethiopia [7,25,31,32]. It has been also observed that central obesity appears to be an independent risk factor for poor glycemic control in T2 DM patients [33]. The possible reason might be the result of insulin resistance brought on by obesity and/or overweight.

Insulin resistance develops as a result of the adipose tissue's increased release of fatty acids, lipids, and other progressive components, which results in uncontrolled hyperglycemia [34]. However, the finding is, contradicts the study conducted in Morocco [27] and Iran [35], which reported no association between poor glycemic control and obesity and overweight in T2 DM patients. The difference may be due to the variation in the study population. In our study, increased SBP is also associated with poor glycemic control in T2 DM patients. Although the causal-effect relationship between Blood Pressure (BP) and glycemic control in DM was not clearly understood yet, studies reported that poor glycemic control is very common in patients with uncontrolled BP [36,37]. On the other hand, poor glycemic control is reported as one of the determinant factors for hypertension or uncontrolled BP in DM patients [38,39]. Furthermore, a longer duration of DM (≥10 years) since diagnosis is also significantly associated with poor glycemic control in this study.

In line with these results, studies done in different parts of Ethiopia revealed that patients with a longer duration of DM were more likely to have poor glycemic control compared to those with less duration [15,31,37,40-42]. The hypothesized mechanism might be due to the chronic and progressive nature of the disease. Insulin secretion will gradually become compromised over time by beta cells, which will also cause an increase in insulin resistance and a rapid drop in insulin secretion [43].

However, the findings of this study have to be interpreted in light of some limitations. First, the study sample size was insufficient and it was a single-center study, it may be difficult to extrapolate the findings to the entire community of T2 DM patients. Second, due to resource limitations, we did not measure the lipid profile of participants, which are additional indicators of metabolic risk factors. Finally, other determinant factors, including treatment adherence and dietary habits of study participants, that could be linked to poor glycemic control were not addressed.

## Conclusion

The majority of study participants have poor glycemic control. Factors such as obesity and/or overweight, systolic hypertension, longer duration of disease, and FBS are independent factors associated with poor glycemic control in T2 DM patients. Therefore, the study emphasizes the significance of managing and preventing both high blood pressure and obesity as well as providing special attention to patients with longer duration to keep the glycemic status within acceptable bounds.

### What is known about this topic

- *The major goal of therapeutic intervention to reduce diabetes-related mortality and morbidity is to maintain a good glucose level;*
- *Estimating glycated hemoglobin (HbA1c) is the standard method for assessing long-term glycemic control;*
- *The majority of studies in Ethiopia use fasting blood sugar (FBS) to estimate the prevalence of glycemic control, which is influenced by a number of factors*.

### What this study adds

- *The prevalence of poor glycemic control is high in the study population;*
- *Metabolic risk factors are independent predictors of poor glycemic control in T2 DM patients*.
